# The CombE-IDMS Alternate Potency Method for H5N1 and H5N8 Cell-Based Vaccines

**DOI:** 10.3390/vaccines11121799

**Published:** 2023-12-01

**Authors:** Matthew P. Donohue, Zhijun Cao, Thomas Bowen, Robert Dickinson, Ying Zhang, Jiang Qian

**Affiliations:** 1 Biopharmaceutical Product Development, CSL Seqirus, Holly Springs, NC 27540, USA; zhijun.cao@seqirus.com (Z.C.); thomas.bowen@seqirus.com (T.B.); ying.zhang@seqirus.com (Y.Z.); 2 CSL Limited, Melbourne, VIC 3000, Australia; robert.dickinson@csl.com.au

**Keywords:** alternate potency, isotope dilution mass spectrometry (IDMS), hemagglutinin (HA), avian influenza, forced degradation

## Abstract

Assaying the potency of inactivated viral influenza vaccines is performed using single radial immunodiffusion, which is the globally accepted release method for potency. Under conditions of a rapidly emerging pandemic, such as the 2009 H1N1 influenza pandemic, a recognized obstacle in the delivery of vaccines to the public is the time needed for the distribution of calibrated SRID reagents (antisera and antigen standards) to vaccine manufacturers. Previously, we first described a novel streamlined MS-based assay, CombE-IDMS, which does not rely on antisera/antibodies or reference antigens, as a potential rapidly deployable alternate potency method through a comparison with SRID on adjuvanted seasonal quadrivalent vaccine cell-based (aQIVc) materials. In this report, we further demonstrate that the CombE-IDMS method can also be applied to measure the potency of pre-pandemic H5N1 and H5N8 monovalent vaccine materials, each subtype both unadjuvanted and adjuvanted, through a forced degradation study. Overall, CombE-IDMS results align with those of the gold standard SRID method on both H5N1 and H5N8 materials under conditions of thermal, pH, oxidative and freeze/thaw stress, lending further evidence for the CombE-IDMS method’s suitability as an alternate assay for potency of both seasonal and pandemic influenza vaccines.

## 1. Introduction

The lack of population-level immunity within humans to avian influenza viruses presents a serious health threat [1]. At any given time, this infectious disease has the potential to cause a pandemic. The transmission of highly pathogenic avian influenza H5N1 and H5N8 viruses is influenced by an expanding global poultry industry [1,2], and the widespread nature [3] of these viruses is concerning based on the associated probability for an antigenic shift to occur. Pandemic preparedness is warranted in order to rapidly respond to demands placed on both health care systems and national economies [4]. Within a preparedness strategy, the most important aspect for controlling the spread of infection is through vaccine allocation and administration [5]. Stockpiling of vaccines remains an important pillar within this strategy; however, as influenza viruses are constantly mutating, a reduced vaccine effectiveness is likely if there is a mismatch between the vaccine and the circulating pandemic strain. This prompts that readiness efforts be made on the part of vaccine manufacturers for production of a strain-matched vaccine after a pandemic outbreak occurs, including development of new technologies which might be applied to aid in production efforts.

Approximately five to six months are needed to deliver approved vaccines of a new influenza strain to the public. A recognized bottleneck in this process is the two-to-three-month timeframe [6] needed for regulatory laboratories to calibrate and distribute the critical reagents, namely antisera and lyophilized antigen standards, used in the single radial immunodiffusion (SRID) assay. Pandemic strains in particular pose a challenge to the reagent development process due to their novelty and the inherent inexperience that comes with producing the materials [7]. Though SRID has been the globally accepted potency release assay for inactivated influenza vaccines since 1978 [8], known limitations in addition to timely reagent production include a narrow dynamic range and low throughput [9]. One of the lessons learned following the 2009 H1N1 pandemic was that an alternate potency assay was needed for a more rapid lot release of the influenza vaccine. An alternate potency assay which is independent of antisera would enhance the rapidity of delivering a vaccine by eliminating the SRID reagent distribution bottleneck.

Essential regulatory laboratories (ERLs) currently use isotope dilution mass spectrometry [10] (IDMS) to assay and calibrate the quantity of hemagglutinin (HA), the main antigen of influenza, in primary liquid standards (PLS, whole inactivated virus material [11]) prior to the PLS being used in SRID as a calibrant for lyophilized antigen standard reagents. IDMS is a validated physicochemical method which is accurate, precise, sensitive and subtype specific [12]. The method is antisera/antibody free and instead uses subtype conserved synthetic peptides as calibration standards; this is advantageous in that the synthetic peptide reagents can be stored by vaccine manufacturers in advance of a pandemic. IDMS, however, is incapable of distinguishing between HA which is antigenic and HA which has lost its structural integrity. To assay for potency, that is, to specifically quantitate antigenic HA conformed to its pre-fusion (pre-F) structure [13], a limited trypsin digestion (LTD) step was included prior to IDMS analysis in previous work using pre-pandemic H7N9 [14] and H5N1 [15] influenza vaccines. Under native conditions, the LTD pretreatment step is designed to specifically digest the susceptible [16] HA1 chain of post-fusion (post-F) and/or stressed conformations of HA, which are conformational populations of lower antigenicity [17] than that of pre-F HA. Pre-F HA is resistant to protease digestion due to its tightly folded conformation. In LTD-IDMS, pretreatment is followed by acetone precipitation of the remaining intact pre-F HA, which is next followed by removal of the soluble tryptic peptides using ethanol washes. The protein pellet is then resuspended, and finally quantitation of the pre-F HA enriched sample is performed by the remaining IDMS steps of denaturation, analytical tryptic digestion and multiple reaction monitoring (MRM) targeted analysis. The precipitation and washing steps must be performed so that tryptic digests from pretreatment do not interfere with the targeted analysis of tryptic peptides generated during the analytical digestion step. LTD-IDMS has been shown to be capable of distinguishing between unstressed and chemically or physically stressed HA, i.e., it is stability indicating, and also to correlate with SRID results, making it an attractive candidate as a rapidly deployable alternate potency assay.

Previously, we reported that a drawback to the LTD-IDMS method was its relatively laborious sample preparation steps. To simplify the method, a combination of enzymes (CombE) chymotrypsin and elastase was used instead of trypsin in the pretreatment step [18]. As chymotrypsin and elastase cleave at the C-terminal side of aromatic and small hydrophobic residues, respectively, the pretreatment and analytical tryptic digestion steps are differential in regard to their proteolytic substrates. In this way, CombE pretreatment digests does not interfere in the targeted analysis of tryptic peptides from the analytical digestion step, thereby eliminating the need for the precipitation and washing steps. Applied to a cell-based adjuvanted quadrivalent seasonal influenza drug product material, the CombE-IDMS method was observed to have an overall agreement in trending under forced degradation conditions with LTD-IDMS and importantly with SRID. Here, we extend the CombE-IDMS method’s applicability beyond seasonal influenza strains and quantitate the potency of H5N1 and H5N8 MF59 adjuvanted and unadjuvanted monovalent materials under a range of stressors. Comparable results to those of SRID are observed, demonstrating the method’s capability to assay vaccine potency under conditions of an emerging pandemic while also eliminating the antisera reagent bottleneck.

## 2. Materials and Methods

### 2.1. Influenza Monovalent Vaccines 

The starting materials used were monobulk drug substances. For research purposes only, these were Madine-Darby Canine Kidney cell-based drug substances. Monobulk materials of A/Turkey/turkey/1/2005 (H5N1) and A/Astrakhan/3212/2020 (H5N8) were each diluted to a 200 µg/mL target HA concentration using 1× PBS (Gibco, cat. 20012-027, Grand Island, NY, USA). These unadjuvanted formulations are hereafter referred to as H5N1c and H5N8c. The MF59 adjuvanted cell-based monovalent influenza vaccine materials aH5N1c and aH5N8c were each formulated by diluting A/Turkey/turkey/1/2005 (H5N1) monobulk and A/Astrakhan/3212/2020 (H5N8) monobulk, respectively, using 1× PBS and MF59 to a target HA concentration of 15 µg/mL and 50% MF59 *v*/*v* for the peptide surrogate selection experiment (see Supplementary Information for experimental details), and also to 200 µg/mL and 50% MF59 *v*/*v* for the forced degradation experiment.

### 2.2. Unstressed Control Samples

In total, 500 µL of 1× PBS was added to 750 µL of the unadjuvanted and adjuvanted monovalents formulated to 200 µg/mL HA. These were kept at 5 °C until testing.

### 2.3. Sample Stress by Mild Low pH 6 

To 750 µL of the unadjuvanted and adjuvanted monovalents formulated to a 200 µg/mL HA, 75 µL of a 0.5 M citric acid buffer, pH 6.0 (Alfa Aesar p/n J61815, Haverhill, MA, USA) was added and stored at room temperature for 17 h. The pH was measured to be 6.0 using a semi-micro pH electrode (Thermo Fisher Scientific, cat. 8102BN, Waltham, MA, USA) connected to an Orion 4 Star digital pH meter (Thermo Fisher Scientific, cat. 1115003). For 17 h, the materials were kept under this pH condition. They were then neutralized to pH 7.2 by the addition of 25 µL of a 1 M Tris-HCl, pH 8.0 (Thermo Fisher Scientific, cat. J22638-AP). Next, the pH was slightly dropped to 7.0 by the addition of 100 µL of 10× PBS (Gibco p/n 70013-032). Finally, 300 µL of 1× PBS was added, and the samples were placed at a 5 °C storage until it was time to test. Immediately prior to testing by SRID, 300 µL of the stressed materials was diluted into 600 µL of 1× PBS to bring the HA concentration into the working range of the assay. Stressed materials remained undiluted prior to assaying by CombE-IDMS.

### 2.4. Sample Stress under Elevated pH Conditions

The pH of 750 µL of the unadjuvanted and adjuvanted monovalents formulated to a 200 µg/mL HA was raised to 9.5 by the addition of a 50 µL 1 M Tris-HCl, pH 9.8 (Quality Biological, cat. 351-176-131, Gaithersburg, MD, USA). For 17 h, at room temperature, the materials were kept at this pH. Neutralization to pH 7.2 was performed by the addition of 350 µL of a 0.5 M citrate buffer (Alfa Aesar p/n J61815). A total of 100 µL of 10× PBS was then added, and the pH slightly decreased to 7.0. A total of 300 µL of 1× PBS was finally added, and these samples were placed at 5 °C until testing was executed. Immediately prior to testing by SRID, 300 µL of the stressed materials was diluted into 600 µL of 1× PBS to bring the HA concentration into the working range of the assay. Stressed materials remained undiluted prior to assaying by CombE-IDMS.

### 2.5. Sample Stress by Temperature

In total, 750 µL of the unadjuvanted and adjuvanted monovalents formulated to a 200 µg/mL HA were incubated at either room temperature or at 37 °C or at 56 °C in closed chambers, for 17 h at each temperature. Materials were then diluted using a 500 µL 1× PBS and stored at 5 °C until testing. A total of 300 µL of the materials incubated at room temperature and at 37 °C was diluted into 600 µL of 1× PBS to bring the HA concentration into the working range of the assay, while materials incubated at 56 °C remained undiluted prior to assaying by SRID. All temperature stressed materials remained undiluted prior to assaying by CombE-IDMS. 

### 2.6. Oxidative Stress

A total of 12.5 µL of H_2_O_2_ (30% *v*/*v*) (Supelco, cat. 1.07298.0250, Bellefonte, PA, USA) kept on ice was added to 750 µL of the unadjuvanted and adjuvanted monovalents formulated to 200 µg/mL HA. The final concentration of H_2_O_2_ was 0.5%. For 17 h, these samples were kept at room temperature in the dark. Following this incubation period, 487.5 µL of 1× PBS was added. Immediately prior to testing by SRID, 300 µL of the stressed materials was diluted into a 600 µL of 1× PBS to bring the HA concentration into the working range of the assay. Stressed materials remained undiluted prior to assaying by CombE-IDMS. 

### 2.7. Sample Stress by Freeze/Thaw 

A total of 750 µL of unadjuvanted and adjuvanted monovalents formulated to a 200 µg/mL HA were diluted into 500 µL of 1× PBS and stored overnight in a −80 °C freezer. Materials were then moved from the freezer and allowed thawing under ambient room temperature conditions. Thawed samples were stored at 5 °C until testing.

### 2.8. Sample Stress by Low pH 3.5 

To 750 µL of the unadjuvanted and adjuvanted monovalents formulated to a 200 µg/mL HA, 83 µL of a 0.5 M citric acid buffer, pH 3.0 (Alfa Aesar p/n J61391) was added and stored at room temperature for 20 min. The pH readout was 3.5. To neutralize, 181 µL of a 1 M Tris-HCl, pH 8.5 (Thermo Fisher Scientific, cat. J61038.AP) was added, and the pH was 7.2. Finally, for volumetric normalization, 236 µL of 1× PBS was added. At this point, the pH was measured to be 7.0, and samples were refrigerated at 5 °C. 

### 2.9. Combination of Enzymes (CombE) 

The CombE pretreatment protocol was described in Qian et al. [18]. Importantly, 20 µL of each sample was incubated at 37 °C for two hours with chymotrypsin (Promega, cat. V1061, Madison, WI, USA) and elastase (Promega, cat. V1891) at ratio of 1:1:2, chymotrypsin:elastase:HA, the same ratio used previously on seasonal quadrivalent formulations.

### 2.10. SRID Reagents 

SRID reference reagents for A/Turkey/turkey/01/2005 NIBRG-23 (H5N1) were sheep polyclonal heterologous [19] reference antiserum A/Indonesia/05/2005 IBCDC-RG2 (H5N1) lot AS406 provided by the Therapeutic Goods Administration (TGA) (PO Box 100, Woden ACT 2606, Canberra, Australia) and reference antigen A/Turkey/Turkey/1/2005 NIBRG-23 (H5N1) lot H5-Ag-1510 provided by the Center for Biologics Evaluation and Research (CBER) (10903 New Hampshire Avenue, Silver Spring, MD 20993-0002). SRID reference reagents for A/Astrakhan/3212/2020 SEQHS_SYS22 (H5N8) were sheep polyclonal heterologous reference antiserum A/Indonesia/05/2005 IBCDC-RG2 (H5N1) lot AS406 provided by the Therapeutic Goods Administration (TGA) (PO Box 100, Woden ACT 2606, Canberra, Australia) and reference antigen A/Astrakhan/3212/2020 SEQHS_SYS22 (H5N8) lot 354398 provided by the CSL Seqirus (475 Green Oaks Parkway, Holly Springs, NC 27540).

### 2.11. SRID and IDMS Protocols 

SRID and IDMS protocols were described in Qian et al. [18]. For IDMS, the HPLC gradient was slightly modified in this work: namely, it was initialized at a 5% MPB from 0 to 1 min, increased to a 10% MPB from 1 to 1.5 min, increased to a 30% MPB from 1.5 to 7 min, ramped to a 98% MPB from 7 to 7.1 min and equilibrated until 8.1 min, and finally ramped down to a 5% MPB from 8.1 to 8.2 min and equilibrated until the end of the run at 11 min. 

### 2.12. CombE-IDMS Method Prequalification

Following the forced degradation experiments, the CombE-IDMS method was prequalified for precision, linearity and accuracy characteristics. These assessments are described within Supplementary Information. For routine testing of the method, a suggested control sample is also described.

## 3. Results

### Assay Stability Indication through a Forced Degradation Study

In order to assess CombE-IDMS as a stability indicating assay, samples were subjected to forced degradation conditions including (1). Thermal stressors of 56 °C, 37 °C and room temperature (RT) (17 h incubation for all temperatures); (2). A 0.5% *v*/*v* H_2_O_2_-induced oxidation (17 h incubation); (3). Mild–low-pH 6 treatment (17 h incubation prior to neutralization); (4). Very low-pH 3.5 treatment (20 min incubation prior to neutralization) (5). Mild–high-pH 9.5 treatment (17 h incubation prior to neutralization); (6). A 1× freeze/thaw (F/T) cycle. CombE-IDMS results were compared to those of SRID to assess the amount of antigenic HA quantitated. 

Starting materials used in the study were H5N1c and H5N8c formulated to a target concentration of 200 µg/mL. Moreover, the formulations were either adjuvanted at 50% MF59 *v*/*v* or they were unadjuvanted to evaluate the impact of MF59 within the sample matrix. Based on the designed volumetric normalization of all samples, the unstressed control sample was diluted to 120 µg/mL using 1× PBS. To bring the materials into the working range of the SRID assay, the control sample and all stressed materials were further diluted by a factor of three using 1× PBS with the exceptions of pH 3.5, 56 °C incubation and 1× F/T samples, in which case it was expected that the stress itself would decrease the absolute amount of potent HA into the working range of the assay. In this way, the impact of these stressors can be better quantitated. The dynamic range of IDMS is much wider than that of SRID, so for evaluation by CombE-IDMS, all stressed samples remained neat and undiluted.

**Figure 1:**
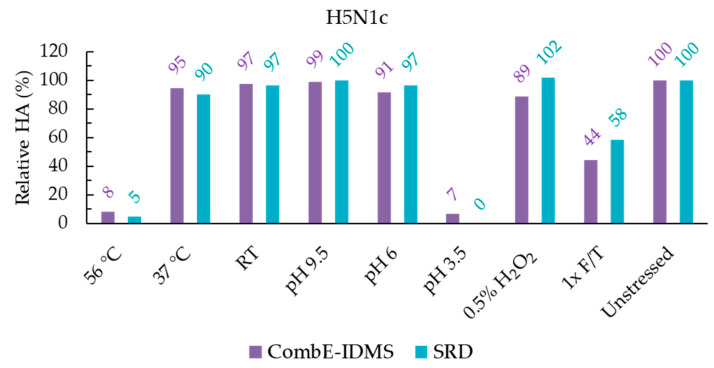

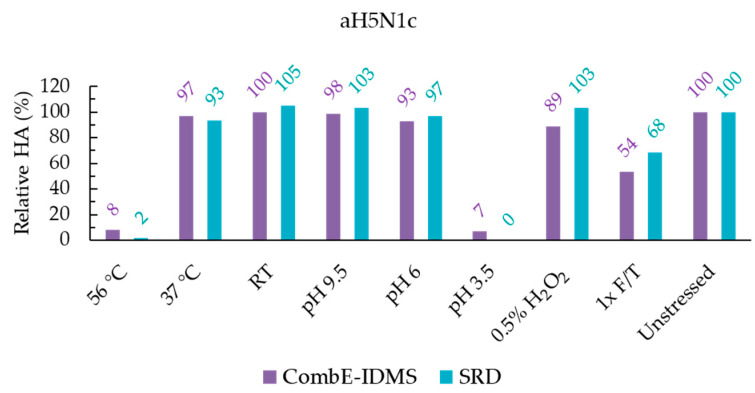
Reportable potency results of H5N1c (**top**) and aH5N1c (**bottom**) by CombE-IDMS and SRID. All results were normalized to the unstressed material. By SRID, no rings were observed following the pH 3.5 stress.

**Figure 2:**
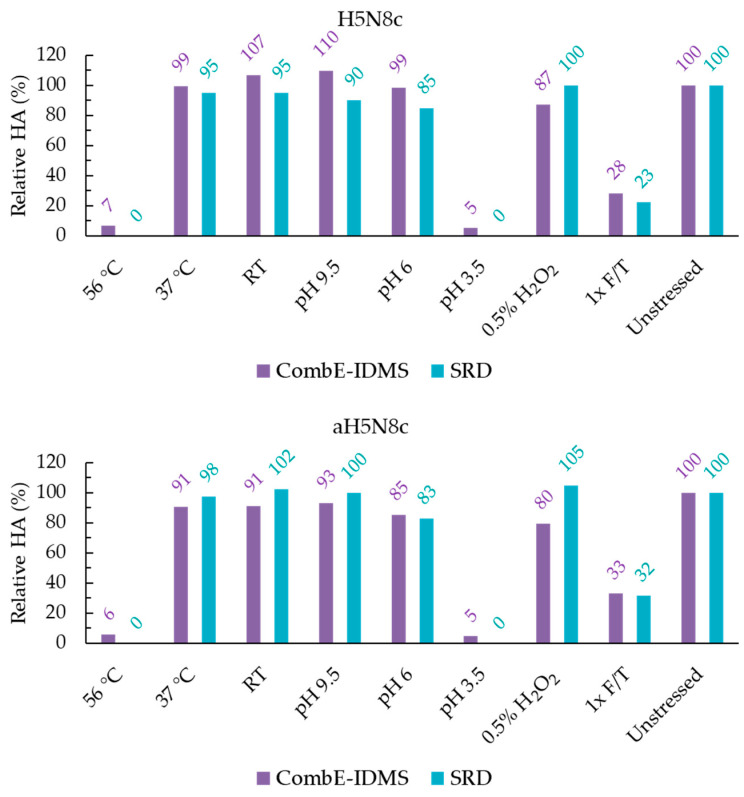
Reportable potency results of H5N8c (**top**) and aH5N8c (**bottom**) by CombE-IDMS and SRID. All results were normalized to the unstressed material. By SRID, no rings were observed following the 56 °C and pH 3.5 stresses.

As concluded from the peptide selection experiments (see Supplementary Information for experimental details and discussions), a 1:1:2 ratio of chymotrypsin:elastase:HA was used during CombE pretreatment. It is worth noting that the same ratio was determined to be optimal in testing seasonal aQIVc vaccine candidates, underscoring the robustness of the sample pretreatment in the CombE-IDMS assay. H5N1 and H5N8 results normalized to unstressed control are shown in Figure 1 and Figure 2, respectively. For what would be considered a reportable H5 potency of CombE-IDMS by monitoring the peptide LVLATGLR, a comparison to SRID potency is displayed to evaluate a correlation between the methods. 

## 4. Discussion

CombE-IDMS is a physicochemical method designed specifically to quantitate properly folded and antigenic HA. A primary advantage of the method is that synthetic peptides are used as calibrators for absolute quantitation of potent HA, and these peptide standards may be purchased and stocked by vaccine manufactures at any point in advance of an influenza pandemic, thereby circumventing the wait time needed to acquire antisera and reference antigen reagents used in the SRID method. We demonstrate in the selection of the surrogate peptide, LVLATGLR, monitored to report potency (see the Supplementary Information for experimental details and results) that the method meets the following criteria: (1) it does not detect post-F HA, as LVLATGLR is maximally digested by CombE pre-treatment of low-pH stressed HA and (2) applying CombE pretreatment to unstressed material generates highly comparable (within 5%) results relative to the total amount of H5, measured by IDMS, within the sample. The latter met criterion indicates LVLATLGR of pre-F HA, which is presumed to comprise nearly the total amount H5 in the unstressed material, is conformed to be resistant to CombE pretreatment and remains contiguous for quantitation by MRM. For this alternate potency method, LVLATGLR was dually concluded to be the sole peptide used to quantitate both subtypes H5N1 and H5N8, lending evidence that the peptide may be generally monitored across H5Nx strains, though more strains need to be tested to further support this. Moreover, the peptide is highly conserved across H5Nx strains based on bioinformatic searches of sequence databases; for example, within the Influenza Virus Resource [20], 3977 out of 4140 deposited H5Nx strains contained (K/R)LVLATGLRX (where X is any amino acid but proline) within full-length HA, a roughly 96.1% conservancy of the tryptic peptide. This conservancy is a critical attribute for stockpiling synthetic LVLATGLR peptide reagents in advance of a pandemic owing to the high probability of the peptide being found in a novel strain.

### 4.1. Thermal Stress

Cold chain deviations were simulated at three separate storage temperatures to assess the impacts on potency. After a 17 h storage at room temperature and 37 °C, HA potency was minimally impacted by either method. Conversely, storage at 56° C resulted in nearly the entire population of HA proteins being degraded and rendered undetectable by SRID, which aligned with CombE-IDMS results. A temperature of 56 °C is likely close to the melting temperature of HA [21] and likely causes denaturation of epitopes and the targeted IDMS peptide.

### 4.2. pH Stress

A common means to induce deamidation on the side chains of either asparagine or glutamine residues is to subject the sample to a high-pH environment. A 17 h incubation at the elevated pH of 9.5 resulted in minimal impact on potency on all materials by both methods. If deamidation was induced somewhere on the protein by pH 9.5 stress, it ostensibly neither modulated the epitope conformations to which the polyclonal anti-HA antibodies bind nor conformed HA to a manner which increases its protease susceptibility.

A mildly acidic pH 6 was also investigated as a stressor. The release pH specification of bulk MF59 is between 6 and 7. Simulating the moment during formulation in which HA first contacts MF59 in its most acidic state was investigated by this stress. The H5N1 materials showed little impact on the potency of either method using pH 6 stress. The unadjuvanted and adjuvanted H5N8 materials assayed by SRID, however, displayed a minor drop in potency to 85% and 83% of the unstressed material, respectively. For the aH5N8c material, CombE-IDMS (85%) also observed this minor drop and agreed well with SRID. For the unadjuvanted H5N8c material under pH 6 stress, CombE-IDMS did not observe a minor drop but remained consistent with the unstressed material at a 99% potency. It is yet unclear why for H5N8c specifically a minor difference in results between methods is observed. One possible reason is the slight underestimation of potency, at 114 µg/mL, of H5N8c by CombE-IDMS to its target formulation of 120 µg/mL in the unstressed material (see Table 1 for [HA] results in µg/mL), though this still is not enough to reconcile a roughly 15% potency decrease. Though the potency drop in question is minor, further investigation is planned to better understand the discrepancy. 

Very low-pH 3.5 treatment resulted in no rings observed by SRID in all materials. Very low potency results, less than or equal to 7% relative to the unstressed sample for all materials tested, were found by CombE-IDMS. Here, inter-method results agreed to indicate a significant-to-complete loss of potent HA through conversion into the post-F conformation, a phenomenon which is well established [13,22].

### 4.3. Oxidative Stress

Hydrogen peroxide is known to cause methionine oxidations in proteins. As a result, the conformation of a protein may be altered and therefore rendered more susceptible to digestion by proteases [23]. By CombE-IDMS, a minor loss of 11% potency of HA by a 0.5% H_2_O_2_ pretreatment was observed for both H5N1c and aH5N1c materials, while by SRID, there appeared to be no impact. Similarly, for H5N8c and aH5N8c, by CombE-IDMS, a loss of 13% and 21% was observed, respectively, while by SRID, the potency appeared to be unimpacted. Here, it is feasible that a subpopulation of HAs undergoes minor conformational changes near the fusion domain in the vicinity of the surrogate peptide LVLATGLR and renders that peptide susceptible to digestion, while for SRID, the epitopes remain recognizable by the polyclonal antibodies within the antisera.

### 4.4. Freeze Thaw Stress

Freeze/thaw (F/T) cycles are generally avoided in storing influenza cell culture vaccine materials because the buffer pH could be dramatically altered during the F/T process [24] and subsequently impact the conformation of HA. For the H5N1 materials, potency by SRID resulted in slightly lesser degradation (58% and 68% of the unstressed control for the unadjuvanted and adjuvanted formulation, respectively) than potency measured by CombE-IDMS (resulting in 44% and 54% of the unstressed control for the unadjuvanted and adjuvanted formulations, respectively). For H5N8 materials, excellent agreement in loss of potency was observed between CombE-IDMS and SRID. For unadjuvanted H5N8c, SRID potency was 23% relative to the control, while that of CombE-IDMS was 28% relative to the control, and for adjuvanted aH5N8c SRID, potency was 32% relative to the control while CombE-IDMS was 33%. As an interesting side note, MF59 appeared to mildly protect the HA from F/T degradation, as a higher quantity of HA was observed by both methods in the adjuvanted material relative to the unadjuvanted material.

In summary, for adjuvanted and unadjuvanted H5N1 and H5N8 materials, CombE-IDMS correlated well with SRID under the two conditions which resulted in a total loss of potency (rings were not observed), i.e., a 56 °C thermal stress and pH 3.5 stress. The methods were also correlated under the condition which resulted in a moderate loss of potency, namely 1× F/T. Storage at 37 °C, room temperature storage and pH 9.5 had little impact on the potency measured by either method. Mildly acidic pH 6 stress resulted in good agreement between methods for all materials with the exception of H5N8c, in which SRID displayed a 15% loss in potency while no loss was observed by CombE-IDMS. For H5N8 in particular, CombE-IDMS appeared slightly more sensitive to a minor potency loss than SRID under the 0.5% H_2_O_2_ treatment; 87% and 80% potencies of the unstressed control by CombE-IDMS for unadjuvanted and adjuvanted formulations were observed, respectively, while 100% and 105% potencies of the unstressed controls by SRID for unadjuvanted and adjuvanted formulations were noted, respectively. This perhaps resulted from a minor conformational change in the microenvironment around the LVLATGLR peptide, rendering a small population of that sequence susceptible to protease cleavage, while the HA epitopes recognized by SRID antisera is not impacted by 0.5% H_2_O_2_ oxidative stress. H5N1 exhibited a minor discrepancy between methods under oxidative stress, in which CombE-IDMS resulted in an 89% relative potency for both adjuvanted and unadjuvanted formulations, whereas SRID did not result in any difference from its control sample. Though slight differences in the magnitude of results were observed between methods, the overall degradation trend by CombE-IDMS correlates with SRID for stress conditions which induce significant, moderate, minor and stable potencies relative to unstressed H5N1 and H5N8 materials.

Absolute potency also agreed well between methods for H5N8 materials as shown in Table 1. These reportable values are critical for CombE-IDMS to be considered as an alternate method for SRID. For H5N1, absolute results were not consistent with SRID (data not shown). Lyophilized antigen standards for this material date to 2015; see Morgenstern et al. [14] for explanatory discussions regarding discrepancies in absolute results between methods dependent on the calibration technique of the standards. 

Moreover, no evidence of MF59 interference was detected, indicating the CombE-IDMS method may be applied to adjuvanted materials. And finally, though forced degradation studies are critical preliminary evaluations of stability indication capable of being executed in a short period of time, our current ongoing efforts are focused on the analysis of potency trending by CombE-IDMS under real-time long term (2–8 °C) and accelerated (23–27 °C/55–65% relative humidity) storage, which are more clinically relevant conditions.

## 5. Conclusions

The global numbers of avian infections and deaths from highly pathogenic influenza H5 viruses, including H5N1 and H5N8 subtypes, have risen in recent years [25]. Though H5N1 is not known to spread well between mammals, the subtype was detected in intensively farmed minks on a Spanish farm in 2022, resulting in the culling of more than 50,000 animals [26] and prompting concerns about the potential for interspecies transmission of the virus into humans [27]. Recognition of the threat of an influenza pandemic originating from an avian H5Nx strain has resulted in preparedness measures taken by governments, global health agencies and vaccine manufacturers, which in part include the search for vaccine alternate potency assays. Recently, we described the antisera/antibody free CombE-IDMS assay applied to cell-based adjuvanted seasonal quadrivalent influenza vaccine material [18]. Here, we applied the method to the study of cell-based H5N1 and H5N8 monovalent materials. Method development first involved the identification of a potency surrogate peptide susceptible to CombE pretreatment digestion in the post-F HA conformation while resistant to digestion in the pre-F HA conformation through optimization of the CombE:HA ratio using low-pH stressed and unstressed materials, respectively. Across both subtypes, the HA1 peptide LVLATGLR met the criteria as a potency surrogate (see Supplementary Information for results). Subsequently, we demonstrated that under forced degradation conditions, the CombE-IDMS method can provide results comparable to the gold standard SRID potency assay. The method continues to provide encouraging performance as a rapid, QC-friendly alternate potency assay for influenza vaccines. 

## Figures and Tables

**Table 1:** Absolute potency results in µg/mL by CombE-IDMS and SRID of H5N8 A/Astrakhan/3212/2020 in the forced degradation study.

Material	Stress Condition	[HA] by CombE-IDMS (µg/mL)	[HA] by SRID (µg/mL)
H5N8c	56 °C	8	NR *
	37 °C	114	114
	RT	122	114
	pH 9.5	126	108
	pH 6	113	102
	pH 3.5	6	NR
	0.5% H_2_O_2_	100	120
	1 × F/T	32	27
	Unstressed	114	120
aH5N8c	56 °C	7	NR
	37 °C	111	120
	RT	112	126
	pH 9.5	114	123
	pH 6	105	102
	pH 3.5	6	NR
	0.5% H_2_O_2_	98	129
	1 × F/T	41	39
	Unstressed	123	123

* NR designates no ring was detected.

## Data Availability

Data are contained within the article and Supplementary Materials.

## Supplementary Materials

The following supporting information can be downloaded at: https://www.mdpi.com/article/10.3390/vaccines11121799/s1, Figure S1: Amino acid sequences of A/Turkey/turkey/1/2005 and A/Astrakhan/3212/2020; Figure S2: aH5N1c candidate peptide responses to CombE pretreatment; Figure S3: aH5N8c candidate peptide responses to CombE pretreatment; Figure S4: Prefusion monomeric structure of H5 in A/Turkey/turkey/1/2005; Figure S5: Reproducibility of the CombE F/T aH5N8c assay control sample; Figure S6: CombE-IDMS assay linearity; Figure S7: CombE-IDMS accuracy to formulation targets.; Table S1: MRM transitions monitored for the LVLATGLR peptide.
