# Supplementary material for: The CombE-IDMS Alternate Potency Method for H5N1 and H5N8 Cell-Based Vaccines
Source: Vaccines (Basel). 2023 Dec 1;11(12):1799. doi: 10.3390/vaccines11121799 (PMC10747648; doi:10.3390/vaccines11121799)
Supplement: Supplementary file 1 [file vaccines-11-01799-s001.zip › vaccines-2688704-supplementary.pdf]

Supplementary Information

The CombE-IDMS alternate potency method for H5N1 and H5N8 cell-based vaccines

Combination of Enzymes (CombE) performed in peptide surrogate selection study.

See the main text for the details of formulating the starting aH5N1c and aH5N8c materials used in this study. Briefly, monobulks of each subtype were formulated to a target HA concentration of 15 µg/mL and included 50% MF59 v/v. For low pH stress, to 540 µL of formulated material, 60 µL of 0.5 M citrate buffer pH 3.0 (Alfa Aesar p/n J61391) was added, dropping the pH to 3.5, and then incubated at room temperature for 20 minutes. For the unstressed samples, to 540 µL of material, 60 µL of water was added and then incubated at room temperature for 20 minutes. For neutralization of the low pH stressed material 130 µL of 1 M Tris pH 8.5 (Alfa Aesar cat. J61038) was added, while for the unstressed samples 130 µL of 1X PBS (Gibco, cat. 20012-027) was added. The final pH of both the stressed and unstressed materials was 7.1.

A range of CombE:HA w/w ratios, as well as mass ratios of chymotrypsin to elastase within the CombE mixture itself at given CombE:HA ratios, were applied to the low pH stressed and unstressed materials. Chymotrypsin (Promega, cat. V1061) and elastase (Promega, cat. 1891) were each first reconstituted to 1 mg/mL in 50 mM Tris pH 8.5 (Alfa Aesar, p/n J61038). To 20 µL of the neutralized stressed and unstressed materials, 4.5 µL of a mixture of chymotrypsin and elastase at a given concentration and composition, dependent on the target CombE:HA ratio, was added and incubated at 37 °C for two hours in an Eppendorf Thermomixer C (VWR, p/n 89428-662) with Thermotop (FisherSci, p/n 05-412-516) at 300 rpm. The remainder of the preparation, continued at the addition of Rapigest^{TM}, proceeds as described in the main text of Qian et. al.[1] under the section **Combination of Enzymes (CombE) performed in forced degradation study.**

To qualify as an appropriate surrogate for potency, monitored HA1 peptides needed to satisfy two criteria: first, a potency surrogate peptide must be protease susceptible in the low pH stressed materials and maximally digested by CombE and second, that same peptide must remain protease resistant in the unstressed material so that CombE pretreatment will not digest the desirable, antigenic pre-F HA population. Proper identification of such peptides provides potency specificity to the method.

Peptide candidates within HA1, the protease susceptible chain in the post-F HA conformation, were first identified from the primary sequences of the H5Nx strains listed in Table S1 and then monitored by LC-MS/MS MRM to assess their utility as potency surrogates. (Note: HA2 chain peptide candidates were also monitored, however, as expected none displayed the desired potency characteristics, and their results are not shown here for brevity). A key parameter to optimize within the assay was the CombE to HA ratio (w/w) under fixed pre-treatment time. The parameter was optimized by testing pH 3.5 stressed and also un-stressed H5Nx at drug product concentrations by varying: 1) the amount of the total mass of CombE (i.e. the summed masses of chymotrypsin and elastase) and 2) the relative proportions of chymotrypsin and elastase within the CombE mixture. Figures S2-S3 (aH5N1c and aH5N8c materials, respectively) display the optimization results of the monitored HA1 candidate peptides by CombE-IDMS on both conditions (low pH stressed and unstressed) normalized to an unstressed sample without CombE pretreatment (the control which measures the total amount of HA present in the sample), i.e., Figures S2-S3 illustrate the relative extent of pretreatment digestion for a given CombE amount and composition. The integrated native peak area of each sample is divided by its internal standard labeled peak area to

generate a response factor, and each sample’s response factor is normalized to the control’s response factor.

Strain: **A/Turkey/turkey/1/2005**
Subtype: **H5N1**
GISAID ISOLATE ID: **EPI_ISL_10107**

HA1

001 MEKIVLLLAI VSLVKSDQIC IGYHANNSTE QVDTIMEKNV TVTHAQDILE KTHNGKLCDL DGVKPLILRD
071 CSVAGWLLGN PMCDEFLNVP EWSYIVEKIN PANDLCYPGN FNDYEELKHL LSR**INHFEKI QIIPK**SSWSD
141 HEASAGVSSA CPYQGRSSFF RNVVWLIKK**D NAYPTIK**RSY NNTNQEDLLV LWGIHHPNDA EQTRLYQNP
211 TTYISVGTST LNQRLVPKIA TRSKVNGQSG RMEFFWTILK PNDAINFESN GNFIAPENAY KIVKKGDSTI
281 MKSELEYGNC NTKCQTPIGA INSSMPFHNI HPLTIGECPK YVKSSR**LVLA TGLR**NSPQGE RRRKKR

HA2

347 GLFGAIAGFI EGGWQGMVDG WYGYHHSNEQ GSGYAADKES TQKAIDGVTN KVNSIIDKMN TQFEAVGREF
417 NNLERRIENL NKKMEDGFLD VWTYNAELLV LMENERTLDF HDSNVKNLYD KVRLQLRDNA KELGNGCFEF
487 YHRCDNECME SVRNGTYDYP QYSEEARLKR EEISGVKLES IGTYQILSIY STVASSLALA IMVAGLSLWM
557 CSNGSLQCRI CI

Strain: **A/Astrakhan/3212/2020**
Subtype: **H5N8**
GISAID ISOLATE ID: **EPI_ISL_13655141**

HA1

001 MENIVLLLAI VSLVKSDQIC IGYHANNSTE QVDTIMEKNV TVTHAQDILE KTHNGKLCDL NGVKPLILKD
071 CSVAGWLLGN PMCDEFIRVP EWSYIVERAN PANDLCYPGS LNDYEELKHL LSRI**NHFEKI LIIPK**SSWPN
141 HETSLGVSAA CPYQGAPSFF RNVVWLIKK**N DAYPTIK**ISY NNTNREDLLI LWGIHHSNNA EEQTNLYKNP
211 TTYISVGTST LNQRLVPKIA TRSQVNGQRG RMDFFWTILK PDDAIHFESN GNFIAPEYAY KIVKKGDSTI
281 MKSGVEYGHC NTKCQTPVGA INSSMPFHNI HPLTIGECPK YVKSNK**LVLA TGLR**NSPLRE TR

HA2

343 GLFGAXAGFI EGGWQGMVDG WYGYHHSNEQ GSGYAADKES TQKAIDGVTN KVNSIIDKMN TQFEAVGREF
413 NNLERRIENL NKKMEDGFLD VWTYNAELLV LMENERTLDF HDSNVKNLYD KVRLQLRDNA KELGNGCFEF
483 YHKCDNECME SVRNGTYDYP QYSEEARLKR EEISGVKLES IGTYQILSIY STAASSLALA IMMAGLSLWM
553 CSNGSLQCRI CI

Figure S1: Amino acid sequences of A/Turkey/turkey/1/2005 H5N1 (top) and A/Astrakhan/3212/2020 H5N8 (bottom). Subtype and GISAID isolate information is provided in the figure. Sequences are broken by polypeptide chain HA1 and HA2. Candidate HA1 potency surrogate peptides are highlighted.

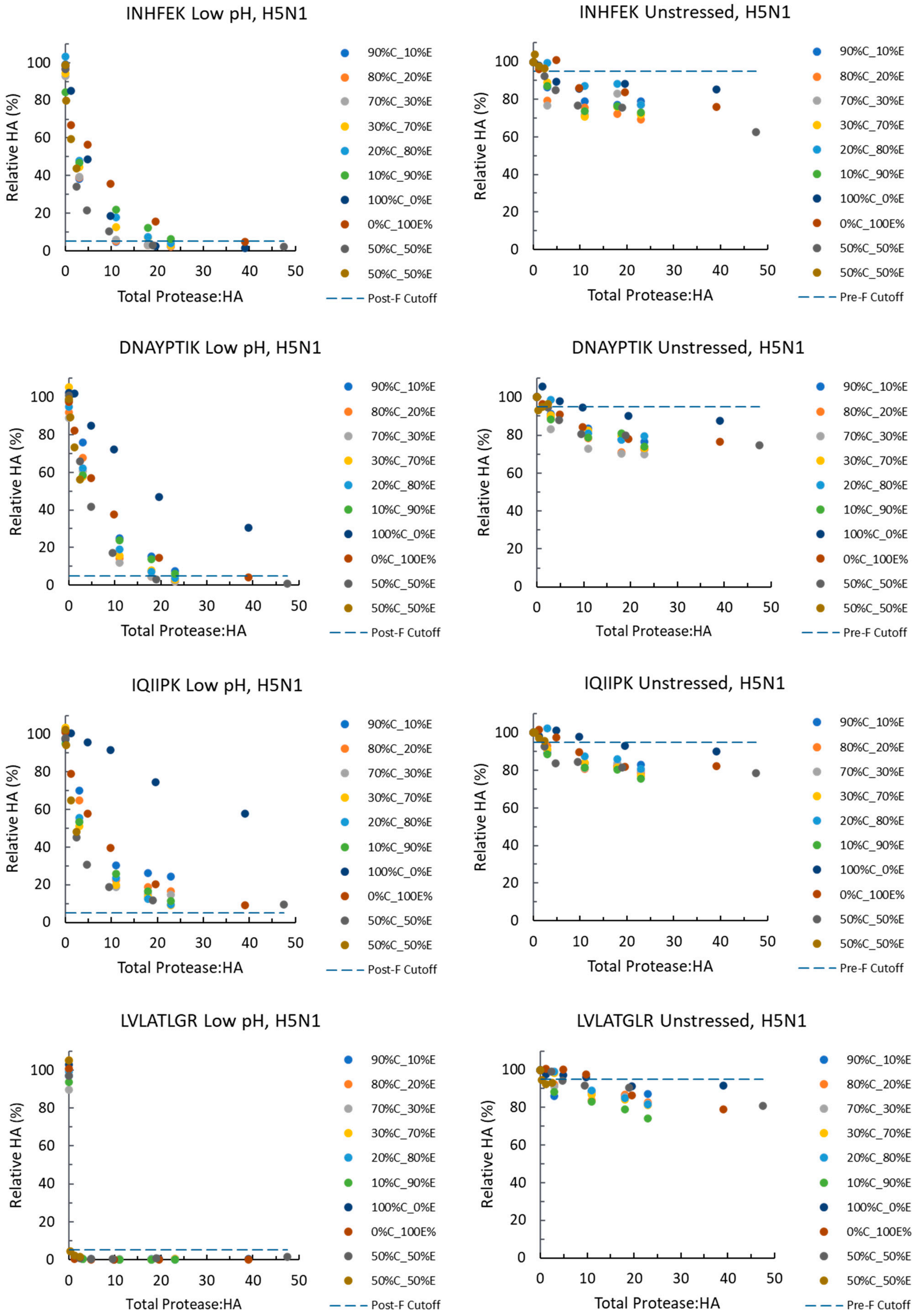

INHFEK Low pH, H5N1
INHFEK Unstressed, H5N1
DNAYPTIK Low pH, H5N1
DNAYPTIK Unstressed, H5N1
IQIIPK Low pH, H5N1
IQIIPK Unstressed, H5N1
LVLATLGR Low pH, H5N1
LVLATGLR Unstressed, H5N1
Relative HA (%)
Total Protease:HA
0
20
40
60
80
100
0
10
20
30
40
50
90%C_10%E
80%C_20%E
70%C_30%E
30%C_70%E
20%C_80%E
10%C_90%E
100%C_0%E
0%C_100E%
50%C_50%E
50%C_50%E
Post-F Cutoff
Pre-F Cutoff

Figure S2. In aH5N1c, the response of HA1 candidate peptides to CombE pretreatment under low pH stressed and unstressed conditions. The legend describes the relative mass proportions of chymotrypsin (C) and elastase (E) within the CombE mixture. All sample response ratios are normalized to that of their control's, which is the unstressed material without CombE-pretreatment, i.e. the total amount of HA present measured by IDMS. A threshold is drawn (dashed blue line) at the 5% level for the low pH stressed condition and at the 95% level for the unstressed condition as a guide to the eye, signifying the desired digestion extent of protease susceptible conformations (i.e. post-fusion HA) and the desired resistance to protease digestion in antigenic conformations (i.e. pre-fusion HA), respectively. The candidate peptide LVLATGLR at relatively low Total Protease:HA ratios is both maximally digested in the low pH stressed condition (data points fall below the Post-F cutoff) and remains undigested in the unstressed condition (data points lie above the Pre-F cutoff), which is the desired peptide behavior for the CombE-IDMS assay.

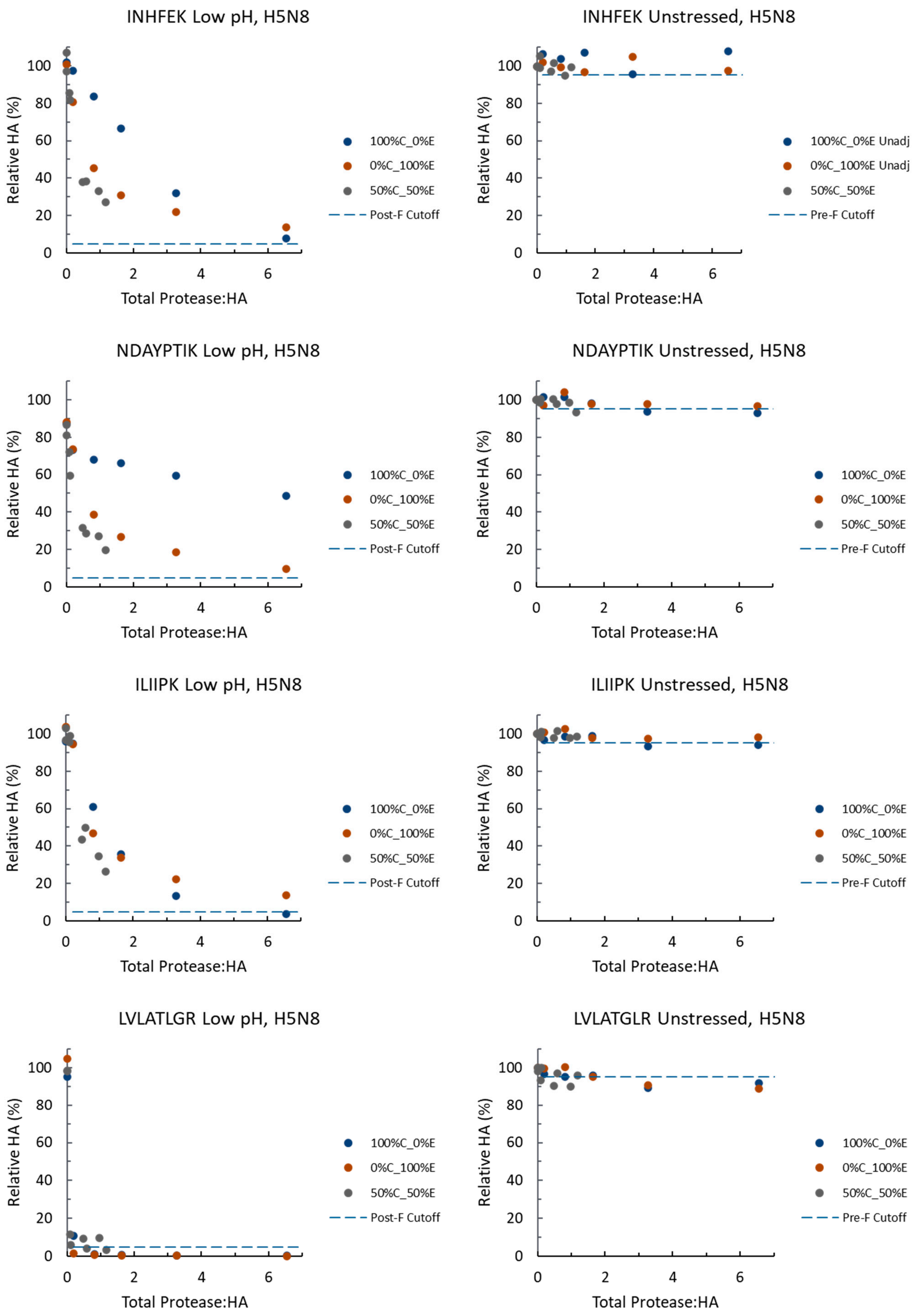
INHFEK Low pH, H5N8
Relative HA (%)
Total Protease:HA
100%C_0%E
0%C_100%E
50%C_50%E
Post-F Cutoff
INHFEK Unstressed, H5N8
Relative HA (%)
Total Protease:HA
100%C_0%E Unadj
0%C_100%E Unadj
50%C_50%E
Pre-F Cutoff
NDAYPTIK Low pH, H5N8
Relative HA (%)
Total Protease:HA
100%C_0%E
0%C_100%E
50%C_50%E
Post-F Cutoff
NDAYPTIK Unstressed, H5N8
Relative HA (%)
Total Protease:HA
100%C_0%E
0%C_100%E
50%C_50%E
Pre-F Cutoff
ILIIPK Low pH, H5N8
Relative HA (%)
Total Protease:HA
100%C_0%E
0%C_100%E
50%C_50%E
Post-F Cutoff
ILIIPK Unstressed, H5N8
Relative HA (%)
Total Protease:HA
100%C_0%E
0%C_100%E
50%C_50%E
Pre-F Cutoff
LVLATLGR Low pH, H5N8
Relative HA (%)
Total Protease:HA
100%C_0%E
0%C_100%E
50%C_50%E
Post-F Cutoff
LVLATGLR Unstressed, H5N8
Relative HA (%)
Total Protease:HA
100%C_0%E
0%C_100%E
50%C_50%E
Pre-F Cutoff

Figure S3. In aH5N8c, the response of HA1 candidate peptides to CombE pretreatment under low pH stressed and unstressed conditions. The legend describes the relative mass proportions of chymotrypsin (C) and elastase (E) within the CombE mixture. All sample response ratios are normalized to that of their control's, which is the unstressed material without CombE-pretreatment, i.e. the total amount of HA present measured by IDMS. A threshold is drawn (dashed blue line) at the 5% level for the low pH stressed condition and at the 95% level for the unstressed condition as a guide to the eye, signifying the desired digestion extent of protease susceptible conformations (i.e. post-fusion HA) and the desired resistance to protease digestion in antigenic conformations (i.e. pre-fusion HA), respectively. The candidate LVLATGLR at relatively low CombE:HA ratios is both fully digested in the low pH stressed condition and remains undigested in the unstressed condition, which is the desired peptide behavior for the CombE-IDMS assay.

In the low pH stressed materials which are representative of the post-F HA conformation of low antigenicity we observe very high proportions of pretreatment proteases are needed to approach the target maximal digestion of 5% for all peptides except LVLATLGR. Focusing on the aH5N1c material in Figure S2, we observe that at very high amounts of CombE the unstressed material (right column) begins to be digested during pretreatment, imposing a maximum limit on the amount of CombE which can be used to ensure that the proteases will not digest the desired pre-F HA population. Of the four peptide candidates, only LVLATGLR is maximally digested in the low pH stressed condition at relatively small CombE:HA ratios while remaining undigested in the unstressed condition. In Figure S3, for aH5N8c, pretreatment digestion of the low pH stressed material does not appear to have been reached at the highest tested CombE:HA ratio for all peptides except LVLATGLR. For both subtypes, the only peptide which definitively displays potency specificity by CombE-IDMS, i.e. the peptide which is degraded by pre-treatment in the low antigenic conformation of HA (post-F, low pH stress) while fully quantitated despite pre-treatment in the immunogenic conformation (pre-F, unstressed) is LVLATGLR. Therefore, this peptide is selected as a surrogate for potency by the CombE-IDMS method as it demonstrates the method's specificity to digest the post-F LVLATGLR while leaving intact its pre-F conformation. LVLATGLR is also highly conserved across H5Nx subtypes including H5N6 strains.

A 1:1:2 w/w/w ratio of chymotrypsin:elatase:HA, at a 50% chymotrypsin 50% elastase composition, is selected based on Figures S2-S3 and on historical data to align with the method's use on aQIVc materials[1]. Monitoring LVLATGLR at the 50% chymotrypsin 50% elastase composition, the dynamic range of the robustness of the protease to substrate ratio is at least 40, from 1:4 CombE:HA (aH5N1c dataset, in which the unstressed material is digested to 95% of the control and the low pH stressed material is digested to 5% of the control) to 10:1 CombE:HA (aH5N1c dataset, in which the unstressed material is digested to 92% of the control and the low pH stressed material is digested to 1% of the control). The pretreatment step of the assay is robust enough using a fixed amount of CombE to accommodate a wide range of HA concentrations to efficiently digest post-F HA while leaving intact pre-F HA. In addition, at the Total Protease:HA ratio = 1, the response of LVLATGLR is further robust over the range of CombE *compositions* (the various proportions of chymotrypsin and elastase tested, ranging from 0% chymotrypsin, 100% elastase through 100% chymotrypsin, 0% elastase) in that low pH treated material is maximally digested while unstressed material remains resistant. To summarize, not only will varying the amounts of CombE relative to HA over the range of 1:4 to 10:1 (w/w), respectively, generate a robust response, but robustness is maintained even with complete omission of one protease or the other. By extension, this robustness would apply to any lot to lot variations in the activity of the proteases.

These optimization experiments emphasize the requirement to carefully select candidate surrogate peptides especially for potency. As such this presents a limitation to the method, both for the development time needed for new influenza subtypes but also in terms of the method's reliability on (in this case) a single surrogate peptide. In the low probability event (< 1% for H5N1 and < 4% for H5N8 based on peptide conservancy bioinformatics search using the Influenza Virus Database managed by NCBI[2]) that a strain under study contains a mutant variant of the peptide then the assay would be incapable of testing such material. SRID or another suitable alternate potency assay would be needed to assay the mutant strain under that scenario.

The possibility that any structural features which might offer an explanation into each peptide's behavior in the optimization experiments was briefly examined. It is perhaps coincidental, but LVLATGLR is the only peptide located in the fusion domain of HA whereas the remaining HA1 peptides are located in proximity to the receptor binding site and the vestigial esterase domain. See Figure S4 for a structural cartoon highlighting HA1 peptides.

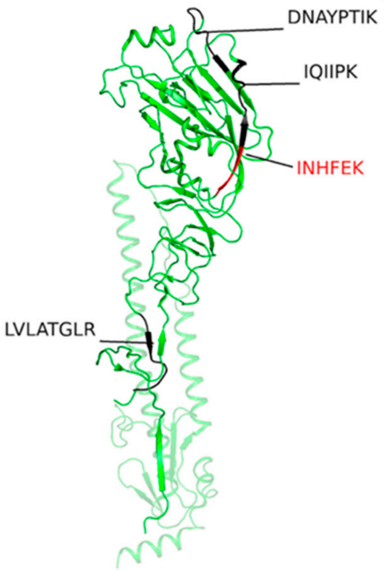

Figure S4: Prefusion monomeric structure of H5 in A/Turkey/turkey/1/2005. The HA1 chain is solid green, the HA2 chain is rendered translucent and each candidate peptide has been labeled. PDB code 5e2z. Note: Post-F HA crystal structures possess extremely truncated HA1 chains due to the necessity to solubilize the membrane protein using bromelain for a high-quality crystal; bromelain digests the susceptible post-F HA1, which is why structural examination of only the Pre-F structure is displayed.

Assay Prequalification: Intermediate precision of Freeze/Thaw Control Sample

An assay control sample is warranted to evaluate the performance of the CombE pre-treatment steps during routine testing. Based on the forced degradation study results within the main text, an intermediate level of susceptibility of the HA1 peptide LVLATGLR to CombE after a freeze/thaw (F/T) cycle was demonstrated, whereas the HA2 peptide EFNNLER is unaffected by this stress (data not shown). Therefore, a F/T aliquot of H5 material serves as both positive control in that CombE digests a population of LVLATGLR and a negative control (CombE does not impact total HA content by monitoring EFNNLER).

aH5N8c formulated to 15 µg/mL and 50% MF59 v/v is used as the assay control, and the material is aliquoted in 30 µL volumes and stored at -80 °C.

Preliminary reproducibility of the F/T control sample subjected to CombE-IDMS was evaluated between two analysts, with each analyst preparing four replicates. Aliquots were moved directly from the -80 °C freezer to the lab bench at room temperature and allowed to thaw for no less than 10 minutes under ambient conditions. 20 µL of the F/T material was then aliquoted into a new tube. Next, 4.5 µL of a CombE working solution of 0.033 µg/µL of chymotrypsin and 0.033 µg/µL of elastase at 1:1:2 chymotrypsin:elastase:HA ratio was added and incubated for 2 hours at 37 °C in an Eppendorf Thermomixer C with a Thermotop at 300 rpm. The remainder of the preparation, continued at the addition of Rapigest™, proceeds as described in the main text of Qian et. al.[1] under the section **Combination of Enzymes (CombE) performed in forced degradation study.** An additional control sample, in which CombE pretreatment was not performed, was included. In place of the working solution of CombE, 4.5 µL of 50 mM Tris pH 8.5 was used; the remainder of the procedure was identical between the control and the F/T samples. See Figure S5 for assay control reproducibility results.

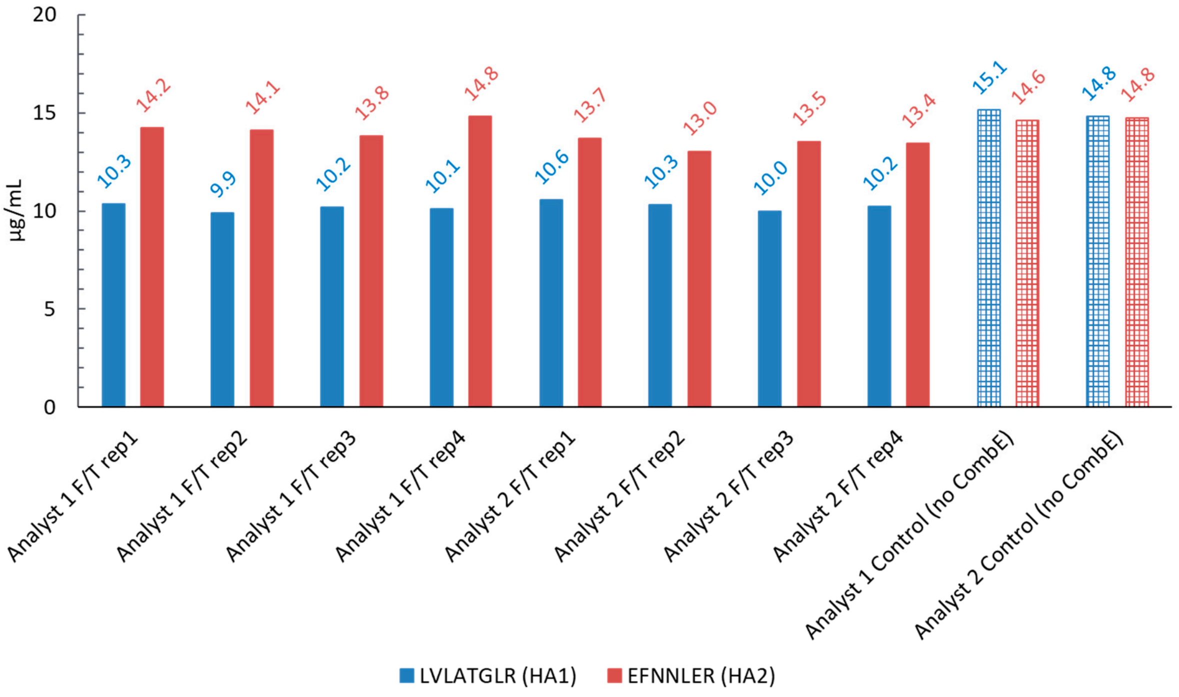

Figure S5: Reproducibility of the CombE F/T aH5N8c assay control sample. Two analysts each performed Combe-IDMS using four replicates, so that $n = 8$ across the two analysts. The potency surrogate LVLATGLR is shown in blue while the total HA surrogate EFNNLER is shown in red. For each analyst, control samples without CombE pretreament are also presented in checkered bars demonstrating the capability of CombE to detect a reduced level of potency by monitoring the LVLATGLR surrogate.

As displayed in Figure S5, the %RSD of the HA2 peptide across samples ($n = 8$) is 3.9% and the %RSD of the HA1 peptide is 2.1%. Moreover, the average ($n = 8$) measured potency by LVLATGLR is 10.2 µg/mL, meaning that approximately 32% of the HA population (based on the 15 µg/mL formulation target, see Control sample results in Figure S5 for total LVLATGLR concentration by omitting pretreatment) has been

degraded by the F/T cycle, an indication that CombE pretreatment is performing as expected and digesting the conformationally compromised population. Therefore, a F/T aliquot of aH5N8c appears to be a suitable CombE assay control to analyze alongside a test article. Freezing at -80 °C also permits long term storage and consistency of the control material by mitigating against any unknown mechanism of potency loss which might occur during refrigerated storage. Note: here, for LVLATGLR the magnitude of digestion by pretreatment differs from that observed in Figures 1-2 of the main text which showed excellent agreement between CombE-IDMS and SRID (a roughly 70% drop in potency by both methods). This is explained by previous observations (data not shown) that the rate of thawing impacts the level of protein degradation. The volume of material used in the forced degradation study in the main text was 1.25 mL compared to the 30 µL used to assess the reproducibility of the assay control, and these differences in volume affect the rate of thawing. Notably, the procedure of thawing the assay control material must be well controlled but as demonstrated here, when followed carefully by analysts it will produce consistent results.

Assay Prequalification: Accuracy and linearity from formulation targeting based on SRID monobulk results.

CombE-IDMS accuracy was assessed from a serial dilution of monobulk H5N8 as the starting material. The monobulk H5N8 SRID measurement resulted in 655 µg/mL HA. The theoretical formulation targets within the serial dilution were calculated from the SRID measurement of the monobulk, so that this assessment can also be an indirect measure of inter-method precision between CombE-IDMS and SRID. All concentration levels were subjected to CombE pretreatment using a fixed 1:1:2 chymotrypsin:elastase:HA ratio and incubated for 2 hours at 37 °C in an Eppendorf Thermomixer C with a Thermotop at 300 rpm. The remainder of the preparation, continued at the addition of Rapigest^{TM}, proceeds as described in the main text of Qian et. al.[1] under the section **Combination of Enzymes (CombE) performed in forced degradation study.** The measured potency at each dilution level by CombE-IDMS is plotted as a function of the theoretical H5 concentration, *i.e*. the formulation target, in Figure S6. The accuracy of the measured potency to that of the target concentration is depicted in Figure S7 for each level in the serial dilution. Percent accuracy is calculated according to the following equation, in which the quotient of the CombE-IDMS measured potency and the theoretical potency is given as a percentage:

$$\%\ \text{Accuracy} = \frac{CombE - IDMS\ measured\ potency}{Theoretical\ potency} * 100$$

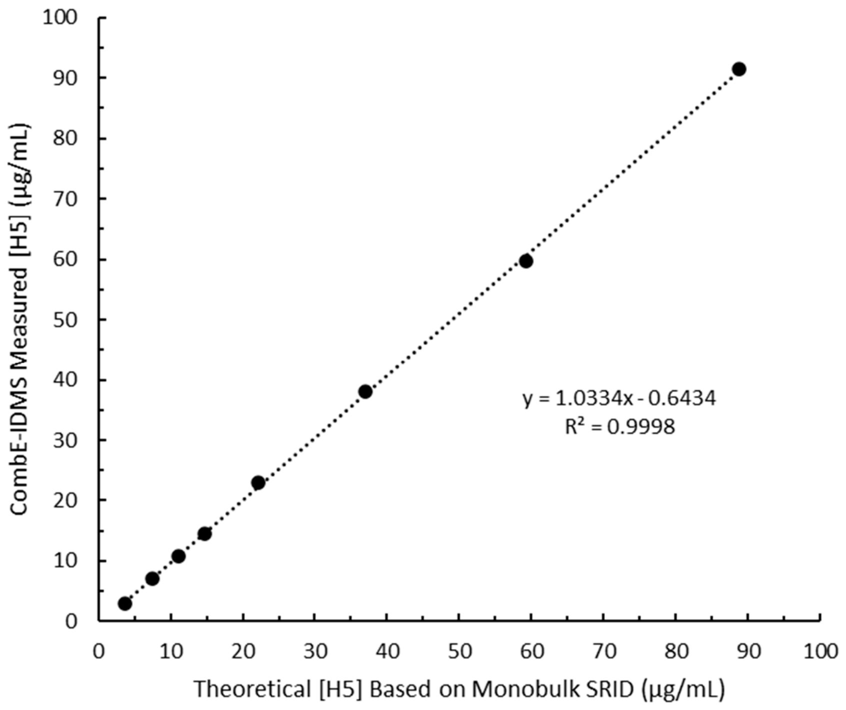

Figure S6: CombE-IDMS assay linearity. Measured potency of a serial dilution of H5N8 by CombE-IDMS as a function of the target concentration, spanning the target formulation range of 3.7 µg/mL to 88.8 µg/mL. A linear regression is fit to the data.

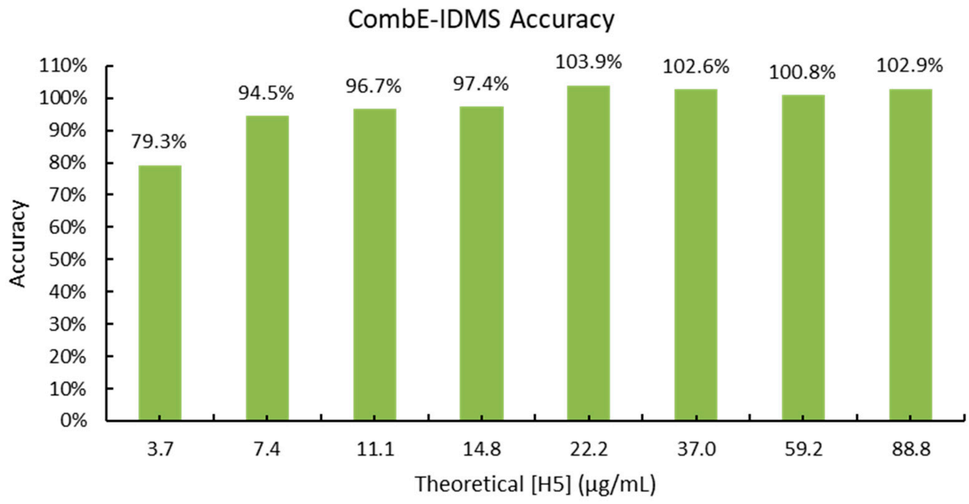

Figure S7: CombE-IDMS accuracy to formulation targets. Formulation targets are calculated from the monobulk SRID value.

The dataset demonstrates the linearity of the method over the range of possible vaccine formulations, though the accuracy does fall off at the lowest tested concentration of 3.7 µg/mL. Finally, the level of

accuracy is also an indirect measure of precision to SRID, as the concentration of the starting monobulk material was itself measure by SRID.

Table S1: MRM Settings for H5-CombE-IDMS monitoring of LVLATGLR

Compound	Retention Time (min)	RT Window (min)	Precursor (m/z)	Product (m/z)	Collision Energy (V)	Min Dwell Time (ms)	RF Lens (V)
LVLATGLR(+2) H5 HA1	5.98	2	421.826	446.272	14.65	40.233	56
LVLATGLR(+2) H5 HA1	5.98	2	421.826	517.309	14.77	40.233	56
LVLATGLR(+2) H5 HA1	5.98	2	421.826	630.393	13.55	40.233	56
LVLATGLR (heavy)(+2) H5 HA1	5.98	2	426.83	456.226	13.09	40.233	56
LVLATGLR (heavy)(+2) H5 HA1	5.98	2	426.83	527.317	14.31	40.233	56
LVLATGLR (heavy)(+2) H5 HA1	5.98	2	426.83	640.401	14.98	40.233	56

References:

1. Qian, J.; Donohue, M.P.; Bowen, T.; Zhang, Y. The CombE-IDMS Assay as an Alternate Potency Method for Adjuvanted Quadrivalent Influenza Vaccines. *Anal. Chem.* **2023**, *95*, 12842–12850. https://doi.org/10.1021/acs.analchem.3c02048

2. Bao, Y.; Bolotov, P.; Dernovoy, D.; Kiryutin, B.; Zaslavsky, L.; Tatusova, T.; Ostell, J.; Lipman, D. The influenza virus resource at the National Center for Biotechnology Information. *J. Virol.* **2008**, *82*, 596–601
